# The writings contain our past, keep our present alive, and give birth to our future

**DOI:** 10.1007/s00259-023-06360-8

**Published:** 2023-08-10

**Authors:** Emilio Bombardieri

**Affiliations:** Nuclear Medicine Unit Humanitas Gavazzeni, Bergamo, Italy

Anyone fortunate enough to have regular access to the pages of a newspaper knows that reading can become a valuable and faithful companion throughout life. A newspaper is a source of knowledge that is easily accessible, that can be consulted at any time of the day, and that always adds something to one’s experience. This statement applies to any type of magazine, be it political, sports, general information, and of course scientific too.

The European Journal of Nuclear Medicine has been one of the most constant references in my professional life, and it happened since 1973 when I started working with professor Buraggi as a young assistant at the Istituto Nazionale dei Tumori in Milan. Month by month this journal helped me to increase my knowledge of Nuclear Medicine and gave me the opportunity to update my culture about innovations and technological advances in our discipline. At the beginning only the copy subscribed to by our Director arrived in our department. Professor Buraggi was jealous and kept the whole collection in his office. Each new issue was of immediate interest to the whole department and there was competition in our group for reading it. I soon came to the conclusion that it was essential to open a personal subscription, in order to have this source of news in my hands and to be able to anticipate a topic of interest to my colleagues. I remember that the direct receipt of a personal hard copy of the journal with my name printed on the package was a great achievement, a kind of emancipation in my carrier as nuclear medicine physician. How many impulses came from discussing the articles, how many ideas about the possibility of translating some recent discoveries or some original approaches into our daily routine, and how many frustrations from the fact that the realisation of some innovations would never become impossible in our realty!

After almost half a century of working in our field, I look back and realise that the world of nuclear medicine has undergone major changes and developments over the past 30 years. Numerous radiopharmaceuticals have been developed, covering a wide range of diseases. In terms of technology we have seen several advances in rapid evolution: the introduction of SPECT and PET, the hybrid imaging revolution, and the introduction of artificial intelligence. Techniques, instruments, procedures, and legislation have all changed radically, including the role nuclear medicine which has become central both as subject of interest for basic and clinical research and for its integration into healthcare system. Of course the journal has also changed in many aspects and contents over the last decades. I remember the first editorial look with the white and blue cover; the replacement with the new elegant colour cover; the change of the title integrated with Molecular Imaging; the modifications of the editorial lay out; the succession of the Editors in Chief Peter Ell, Ignasi Carrio, and Arturo Chiti who marked the history of the journal; and the continuous improvement of the Impact Factor to become the most qualified international journal of Nuclear Medicine.

What makes our journal so special and gives it particular importance? The European Journal of Nuclear Medicine and Molecular Imaging is the official organ of the European Association of Nuclear Medicine (EANM). The great success of the journal parallels the success of the EANM, a major organisation representing the discipline at European level, recognized by public authorities and medical and scientific societies. All members of the EANM are reached by the journal, but it gives space to a much larger community, as it also gathers voices from other scientific associations and brings together virtually all professional groups active in nuclear medicine and related disciplines: radiologists, radiotherapists, physicists, radiochemists, radiopharmacists, and technologists. Both the readers and authors represent a big community engaged in a lively exchange of information, knowledge, and proposals, with an enormous impact on clinical procedures and in particularly in the fields of oncology, cardiology, and neurology.

I have had the pleasure and the honour to contribute to the European Journal of Nuclear Medicine and Molecular Imaging in various ways during my professional career. I have been a member of the editorial board. I had the opportunity to publish several original papers together with my good collaborators. I have written several letters to the Editor, many reviews, and editorials. In particular I would like to remind that in 2003, when I was the coordinator of the EANM Oncology Committee, I proposed and obtained the publication of six EANM Procedure Guidelines for Tumour Imaging, dedicated to the standardisation of different clinical protocols currently used in the clinical routine (breast and bone scintigraphy, ^111^In-pentetreotide, ^67^Ga, ^131^I/^123^I MIG, and ^18^F FDG PET scintigraphy). I invited as co-authors the most prestigious European colleague expert in oncology (R. Baum, A Bishof-Delaloye, J. Buscombe, J. F. Chatal, L. Maffioli, R. Moncayo, L. Mortelmans, S. N. Reske, C. Aktolun). We spend nice time in several intense meetings, and we presented these guidelines as a special educational session of the journal named “blue pages.” This was an example of a joint project between the EANM and the journal to make common clinical protocols available for the international nuclear medicine community.

**Fig. 1:**
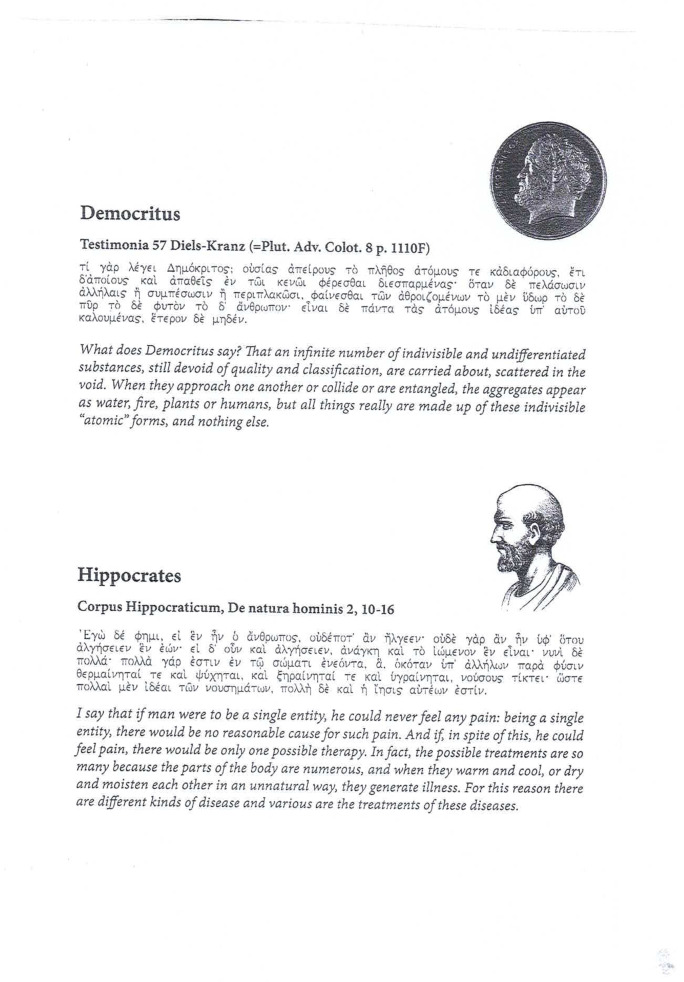
From these concepts which have been modified over the time, a particular medicine, called nuclear medicine, took its first steps and gave life a peculiar discipline distinct from others. There is evidence that every written word has the power to become actions and every action has the power to build up concrete things that can have impact on our lives. In this sense I would like to imagine the role of the European Journal of Nuclear Medicine and Molecular Imaging as an important driving force that fully integrated in the continuous development of nuclear medicine, as a solid tool of information, input, and scientific knowledge

On the occasion of 25th anniversary of the EANM, together with my friend Savas Frangos, as editors of a commemorative book, we wrote that the spirit of our discipline is based on the use of radioactivity according to the rules and ethics of good medical practice (Fig. 1). Democritus and Hippocrates, two great ancient Greek philosophers, formulate these theories that anticipated the discovery of radioactivity and provided the basis for the practice of medicine. If we can say that Nuclear Medicine is more or less 2400 years old, it is because of the writings of Democritus (Testimonia 57 Diels Kranz) (=Plut. Adv. Colot.8p 1110F) and Hippocrates (Corpus Hippocraticus, De natura hominis 2, 10–16).

